# Consistency in reporting sensitive sexual behaviours in Britain: change in reporting bias in the second and third National Surveys of Sexual Attitudes and Lifestyles (Natsal-2 and Natsal-3)

**DOI:** 10.1136/sextrans-2013-051360

**Published:** 2013-11-26

**Authors:** Philip Prah, Andrew J Copas, Catherine H Mercer, Soazig Clifton, Bob Erens, Andrew Phelps, Clare Tanton, Pam Sonnenberg, Wendy Macdowall, Kaye Wellings, Anne M Johnson

**Affiliations:** 1 Research Department of Infection and Population Health, University College London, Mortimer Market Centre, London, UK; 2 NatCen Social Research, London, UK; 3 Health Services Research and Policy, London School of Hygiene and Tropical Medicine, London, UK; 4 Department of Social and Environmental Health Research, London School of Hygiene and Tropical Medicine, London, UK

**Keywords:** Epidemiology (General), Sexual Behaviour, Statistics

## Abstract

**Objectives:**

Britain's second National Survey of Sexual Attitudes and Lifestyles (Natsal-2) was conducted in 1999–2001 and the third (Natsal-3) was conducted in 2010–2012 to update prevalence estimates of sexual behaviours and assess changes over time. We investigated whether there was a change in reporting bias between these two cross-sectional surveys.

**Methods:**

We analysed data from the ‘common birth cohort’ of participants born during 1956–1983, who were eligible to take part in Natsal-2 (n=10 764) and Natsal-3 (n=6907). We compared estimates for outcomes that occurred before Natsal-2 and expected these to be consistent between surveys if no change in reporting bias had occurred.

**Results:**

A greater proportion of non-white men and women were in Natsal-3 consistent with demographic changes in Britain. Reporting behaviours was largely consistent between surveys for men. Fewer women in Natsal-3 reported early first intercourse or having child(ren) before age 20; they were also more likely to report not discussing sex with their parents at age 14. Men and women in Natsal-3 were more likely to report tolerant attitudes towards same-sex partnerships but less tolerance towards unfaithfulness in marriage and one-night-stands.

**Conclusions:**

We found little evidence of change in reporting bias among men since Natsal-2. Among women, a modest change in reporting bias was observed for a small number of experiences, possibly due to changes in participation, social acceptability and methodological differences between surveys. Changes in the reporting of sexual behaviours and attitudes over time observed in the wider Natsal-3 study are therefore likely to largely reflect real changes in the population.

## Introduction

Over the past 20 years, three decennial cross-sectional national probability sample surveys of sexual behaviour, the National Surveys of Sexual Attitudes and Lifestyles (Natsal), have been undertaken in Britain during 1990–1991, 1999–2001 and 2010–2012. Using data from these surveys, we have described changes in reported sexual behaviours in Britain over time.^1^ ^2^ It is important to consider whether these differences in reporting reflect real changes in population behaviour or, at least in part, a change in reporting bias. All surveys are susceptible to different forms of reporting bias (eg, social acceptability and recall bias), which can result in under-reporting or over-reporting of sensitive behaviours;^3^ ^4^ therefore, a change in bias can influence changes in reported behaviours.^5^ A previous study concluded that reporting bias changed between Natsal-1 and Natsal-2, such that participants were somewhat more willing to report socially censured behaviours, specifically same-sex behaviour and early first intercourse, in Natsal-2, attributed to greater social tolerance and improved survey methodology.^6^

Natsal-3 updates prevalence estimates to assess changes in reported behaviour since Natsal-2. Extending previous work,^6^ we present the results of a ‘common birth cohort’ analysis comparing data from the subset of people eligible to take part in both surveys. We assess the difference between Natsal-2 and Natsal-3 in (i) participant demographics, (ii) reporting bias and (iii) attitudes.

## Methods

The Natsal studies are stratified, multistage, probability cross-sectional sample surveys of the general population resident in Britain. In Natsal-2, conducted during 1999–2001, the eligible age range was 16–44, while for Natsal-3, conducted during 2010–2012, the age range was extended to 16–74. Full details of the methods and response calculations are described elsewhere.^1^ ^2^ ^7^ Data from both surveys were weighted to adjust for differential probabilities of selection and a non-response poststratification weight was used to correct for differences in gender, age group and Government Office Region between the Natsal-2 data and the 2001 Census and the Natsal-3 data and the 2011 Census. Question wording and mode of delivery (face-to-face or via computer-assisted self-interviewing (CASI)) were largely identical between surveys. However, reporting pregnancies in Natsal-3 involved a series of questions relating to women's history of pregnancy that were asked in the CASI, while in Natsal-2 a different set of face-to-face questions were asked.

We construct a 27-year ‘common birth cohort’ of participants from the two surveys, born 1956–1983, and therefore of an eligible age to take part in both surveys. We compare the reporting of outcomes that occurred before 1999, thus before Natsal-2. Although participants born between 1956 and 1983 in Natsal-3 are approximately 10 years older than those born in the same years in Natsal-2, aside from recall issues, if there is no change in bias we would expect estimates to be consistent between surveys.

We compare the reporting of demographic characteristics of the common birth cohort before 1999 (last school up to age 16 was single sex, completed continuous full-time education at age 16 and parents were self-employed at age 14) to assess the sample differences, which may confound differences in reporting, as these are less susceptible to reporting bias.

To assess possible changes in willingness to report, we compare the reporting of sensitive behaviours considered more susceptible to bias: first heterosexual intercourse before age 16; same-sex intercourse before 1999; having a child before age 20; not discussing sex with parents around age 14; menarche before age 12 (women only); and, for those who had first heterosexual intercourse before 1999, whether their first partner was more willing, whether they had recently met and non-use of contraception on this first occasion.

Differing social attitudes between surveys may influence willingness to report, so we also compare the reporting of four attitudes at the time of interview: attitudes towards non-exclusivity in marriage, towards one-night-stands, towards same-sex male partnerships and towards same-sex female partnerships.

### Statistical methods

All analyses were performed using complex survey analysis within Stata V.12.1 (StataCorp LP, College Station, Texas, USA) to take account of the stratification, clustering and weighting of the data described above.

We use binary logistic regression, adjusting for year of birth, to produce adjusted odds ratios (AOR) for differences in demographic and behavioural outcomes. We use ordinal logistic regression, adjusted for year of birth, to compare the attitudinal variables. To correct for the possible effect of migration between the two surveys, we also present AORs further adjusted for ethnicity. Demographic differences between the surveys may also explain changes in outcomes, so we also investigate how adjusting for additional demographics found to be different between surveys may influence outcomes.

### Ethics

The Natsal-2 study was approved by the University College Hospital and North Thames Multi-Centre Research Ethics Committee and all the Local Research Ethics Committees in Britain. The Natsal-3 study was approved by the Oxford A Research Ethics Committee (Ref: 09/H0604/27).

## Results

A total of 10 764 of 11 161 participants from Natsal-2 (4596 men and 6168 women) and 6907 of 15 162 from Natsal-3 (2698 men and 4209 women) born between 1956 and 1983 were eligible for these analyses. Table 1 describes the demographic, behavioural and attitudinal comparisons between Natsal-2 and Natsal-3 based on events/conditions occurring before 1999.

**Table 1:** Reporting of demographics, behaviours and attitudinal outcomes for the common birth cohort by gender

	Natsal-2 (%)	Natsal-3 (%)	AOR (95% CI)*		AOR (95% CI)†	
*Men*						
Demographic						
Ethnicity: non-white	8.8	14.6	1.74	(1.45 to 2.09)	−	−
Last school up to age 16 was single-sex	14.8	16.7	1.20	(1.02 to 1.40)	1.16	(0.98 to 1.36)
Completed continuous full-time education at age 16‡	45.4	42.0	0.89	(0.79 to 1.01)	0.94	(0.83 to 1.07)
Parents were self-employed at age 14	18.2	17.6	0.95	(0.82 to 1.11)	0.92	(0.79 to 1.07)
Behaviour						
Had heterosexual intercourse before 16	27.3	26.5	0.96	(0.84 to 1.09)	1.00	(0.88 to 1.14)
Same-sex intercourse before 1999	4.3	4.2	1.01	(0.77 to 1.32)	1.02	(0.78 to 1.34)
Partner was more willing at first heterosexual intercourse§	6.0	4.8	0.79	(0.60 to 1.02)	0.78	(0.60 to 1.02)
Recently met partner at first heterosexual intercourse§¶	20.2	19.0	0.92	(0.79 to 1.07)	0.93	(0.80 to 1.07)
No contraception at first sex§¶	22.5	25.4	1.20	(1.04 to 1.38)	1.18	(1.02 to 1.36)
Had a child before age 20**	3.6	3.4	0.96	(0.69 to 1.33)	0.98	(0.70 to 1.37)
Did not discuss sex with parents around age 14	74.3	75.6	1.09	(0.95 to 1.25)	1.06	(0.92 to 1.21)
Attitudes††						
Non-exclusivity in marriage: always wrong	51.4	56.9	1.17	(1.04 to 1.31)	1.16	(1.03 to 1.30)
One-night-stands: not wrong at all	27.5	19.1	0.60	(0.54 to 0.66)	0.63	(0.57 to 0.70)
Same-sex partnerships (male): not wrong at all	36.4	43.9	1.36	(1.22 to 1.51)	1.48	(1.33 to 1.65)
Same-sex partnerships (female): not wrong at all	41.4	47.9	1.28	(1.15 to 1.42)	1.40	(1.26 to 1.56)
*Women*						
Demographic						
Ethnicity: non-white	8.6	13.5	1.63	(1.37 to 1.93)	–	–
Last school up to age 16 was single-sex	16.6	18.5	1.17	(1.04 to 1.33)	1.12	(0.99 to 1.27)
Completed continuous full-time education at age 16‡	42.6	40.3	0.95	(0.86 to 1.06)	0.99	(0.89 to 1.10)
Parents were self-employed at age 14	19.1	17.5	0.88	(0.77 to 1.00)	0.84	(0.74 to 0.96)
Behaviour						
Had heterosexual intercourse before 16	20.7	18.7	0.85	(0.76 to 0.95)	0.88	(0.79 to 0.98)
Same-sex intercourse before 1999	3.9	3.8	1.00	(0.79 to 1.26)	1.02	(0.81 to 1.29)
Partner was more willing at first heterosexual intercourse§	22.6	17.2	0.71	(0.63 to 0.81)	0.69	(0.61 to 0.78)
Recently met partner at first heterosexual intercourse§¶	9.2	8.1	0.86	(0.72 to 1.03)	0.84	(0.70 to 1.01)
No contraception at first sex§¶	20.6	19.9	0.98	(0.87 to 1.11)	0.95	(0.84 to 1.08)
Had a child before age 20**	14.2	11.5	0.78	(0.66 to 0.92)	0.77	(0.65 to 0.90)
Did not discuss sex with parents at age 14	56.9	63.8	1.37	(1.24 to 1.52)	1.34	(1.21 to 1.48)
Started menstruating before 12	18.1	18.0	0.99	(0.88 to 1.12)	1.00	(0.89 to 1.12)
Attitudes††						
Non-exclusivity in marriage: always wrong	60.0	65.0	1.19	(1.08 to 1.31)	1.17	(1.06 to 1.29)
One-night-stands: not wrong at all	12.5	12.2	0.87	(0.79 to 0.95)	0.91	(0.83 to 0.99)
Same-sex partnerships (male): not wrong at all	52.2	63.5	1.51	(1.38 to 1.67)	1.64	(1.48 to 1.81)
Same-sex partnerships (female): not wrong at all	52.0	63.5	1.52	(1.39 to 1.68)	1.65	(1.49 to 1.82)

*OR for reporting in Natsal-3 relative to Natsal-2, adjusted for year of birth.

†OR adjusted for year of birth and ethnicity (six categories).

‡Participants born before 1983.

§Participants who had first heterosexual intercourse before 1999.

¶Excluded participants whose first sex was forced.

**Participants born before 1980.

††Ordered categorical responses modelled under the assumption of proportional odds.

The proportion of non-white participants increased greatly between surveys, and fewer women (only) reported their parent was self-employed when they were 14 in Natsal-3. However, differences were modest for most of the other demographic factors, especially after additionally adjusting for ethnicity.

With respect to the reporting of sexual behaviours occurring prior to Natsal-2, prevalence estimates were similar for men, and they were only slightly more likely to report not using contraception at first sex in Natsal-3. Women also showed consistent reporting for some behaviours, but, of note, fewer women reported first heterosexual intercourse before age 16 in Natsal-3. Women were also less likely to report having a child before age 20, that their first sexual partner was more willing than themselves, but were more likely to report not discussing sex with their parents around age 14. For men and women, results were unaffected when controlling for ethnicity (table 1), as well as when additionally controlling for whether parents were self-employed at age 14 in women (data not shown).

Among both men and women, reported tolerance of male and female same-sex partnerships was higher in Natsal-3, while tolerance towards non-exclusivity in marriage and one-night-stands was lower.

## Discussion

The reporting of selected sensitive behaviours, occurring before Natsal-2, is largely consistent between Natsal-2 and Natsal-3, especially for men, suggesting that participant's willingness to report has not substantially changed. In women, some differences were in the opposite direction to what might be expected from increased liberal social attitudes. Attitudinal differences were observed for men and women consistent with changes over time between Natsal-2 and Natsal-3.^2^ These could facilitate a change in reporting of behaviours but, unlike our comparisons between Natsal-1 and Natsal-2,^6^ do not appear to have impacted willingness to report sensitive behaviours.

The interpretation of changes seen in a ‘common birth cohort’ would be clearer if the two survey samples were taken from a ‘closed cohort’, but here findings may be influenced by changes in immigration and emigration between 2000 and 2010 (and to a lesser extent mortality), and participation bias in women. The observed changes in ethnic composition are consistent with national population estimates^8^ and can be attributed to changes in the underlying population structure. We note that our findings of reporting behaviours were unaffected after adjusting for ethnicity.

Previously^6^ we observed increased reporting of early sexual intercourse and same-sex behaviour and concluded that greater social acceptability, in line with more relaxed social attitudes, and using CASI may have resulted in a greater willingness to report these behaviours. In the comparison between Natsal-2 and Natsal-3, we have found that in men there was no difference in reporting these behaviours, and among women fewer reported early intercourse. We suggest people are as willing to report these sensitive behaviours such that reporting bias has changed little, if at all. This is perhaps reassuring, at least in the context of same-sex behaviour. Given that we have observed more lenient attitudes towards same-sex partnerships, we might have anticipated greater willingness to report. This consistency in reporting likely reflects the common methodology of the CASI used in both surveys that reassured participants about the privacy of their responses.

Interestingly, women in Natsal-3 were less likely to report having children before age 20 and this is not solely explained by differences in participation. This may also reflect methodological differences between the two surveys as the question wording has changed, as explained above. This may also be an indication of reporting bias due to the changing social attitudes and possibly stigma towards young mothers over time.

In Natsal-3, women were less likely to report that they were less willing than their partner at first intercourse, but were more likely to report not discussing sex with their parents around age 14. These behaviours are based on subjective interpretations and may be influenced by recall bias in Natsal-3 due to the sample being 10 years older. The length of time between event and recall may produce a reassessment of the experience.

Finally, the limited number of factors that could be compared between surveys restricts the extent to which we can assess change in bias across all behaviours measured by the surveys. Despite these limitations and complexities, we conclude that there is consistent reporting (ie, little change in reporting bias) of behaviours between Natsal-2 and Natsal-3. As such, we feel that it is reasonable to generalise our findings to the wider Natsal-3 study and interpret the changes in the reporting of sexual behaviours and attitudes seen during 1999–2001 and 2010–2012^2^ as primarily reflecting real changes in the population.

**Key messages**

- We did a ‘common cohort analysis’ to assess whether willingness to report sensitive behaviours in Britain's National Surveys of Sexual Attitudes and Lifestyles (Natsal) has changed over time.
- We compare participants in Natsal-2 and Natsal-3 born between 1956 and 1983 to examine whether they were consistent as a group in their reporting of behaviours occurring before Natsal-2.
- Some demographic differences exist in the characteristics of participants in each survey, including the proportion of non-white participants, consistent with changing demographic trends in the UK.
- Among men, reporting bias of sensitive behaviours does not seem to have changed.
- Among women, reporting is largely consistent between surveys, with differences likely to be due to a combination of methodological and demographic factors.
- We conclude that changes in reported sexual behaviour observed in the wider Natsal-3 study are likely to reflect real changes in population behaviour.
